# Psychological pathways between leadership behavior and work-family balance: the mediating roles of employee satisfaction and perceived support

**DOI:** 10.3389/fpsyg.2025.1626218

**Published:** 2026-01-12

**Authors:** Pengyuan Wang, Bei Lyu, Qing Yang, Qiang Wan

**Affiliations:** 1 Business School, Dezhou University, Dezhou, China; 2 International Chinese College, Panyapiwat Institute of Management, Nonthaburi, Thailand; 3 College of Social Sciences, Semyung University, Jecheon, Republic of Korea; 4 Graduate School, Hanseo University, Seosan-si, Republic of Korea

**Keywords:** employee satisfaction, human resource management practice, leadership, performance management, work-family balance

## Abstract

This study aims to explore the impact of human resource management practice on employees’ work-family balance (WFB), and examine the role of employee satisfaction (ES) and perceived organizational support (POS) as mediating variables. The structural equation model (SEM) is used to test the research hypothesis by analyzing employees’ questionnaire data of employees in different industries. It is found that leadership has a direct positive impact on employees’ WFB, which is partly realized through the mediating effect of ES. Performance management (PM) practices indirectly promote WFB by enhancing the POS. The SEM’s results show that the standardized path coefficient of leadership on ES is 0.56 (*p* < 0.001), and that of ES on WFB is 0.45 (*p* < 0.001). The standardized path coefficients of POS on WFB and PM on POS are 0.39 and 0.32 (*p* < 0.01). These findings highlight the importance of considering the potential impact on employees’ WFB when designing human resource management strategies. It can be concluded that good leadership and PM practices not only directly improve employees’ WFB but also indirectly contribute to this goal by increasing ES and POS.

## 1 Introduction

Work-family balance (WFB) has become a critical concern for contemporary organizations, as employees increasingly struggle to manage competing demands from work and family domains (Allen et al., 2000; Frone et al., 1992). Achieving optimal WFB is essential not only for employee well-being and life satisfaction but also for organizational outcomes such as productivity, retention, and performance (Bellmann and Hübler, 2021; Wan et al., 2022). Within human resource management (HRM) literature, leadership behavior and performance management practices have emerged as two pivotal organizational mechanisms that shape employees’ work experiences and their capacity to navigate work-family boundaries (Li et al., 2017; Stollberger et al., 2019). Empirical evidence demonstrates that supportive leadership can reduce work–family conflict by enhancing employees’ psychological resources (Park et al., 2023), while effective performance management systems contribute to job satisfaction and overall quality of life (Na-Nan et al., 2020). However, the psychological mechanisms through which these HRM practices jointly influence WFB remain inadequately understood, limiting both theoretical development and practical application.

Despite considerable scholarly attention to work-family dynamics, three critical gaps persist in the extant literature. First, leadership and performance management have predominantly been examined in isolation, with limited exploration of their combined effects on WFB (Daraba et al., 2021; Isa and Indrayati, 2023). Second, the psychological pathways linking HRM practices to WFB outcomes remain underspecified. While employee satisfaction and perceived organizational support are theoretically relevant mediating mechanisms, their specific roles in the HRM-WFB relationship have not been systematically investigated (Hauff et al., 2022; Wayne et al., 2020). Third, existing research has insufficiently integrated boundary theory and conservation of resources (COR) theory to explain how organizational practices simultaneously influence boundary management capacity and psychological resource accumulation. These theoretical and empirical gaps constrain both scholarly understanding and organizational capacity to design evidence-based interventions.

This study addresses these gaps by examining how leadership behavior and performance management practices influence work-family balance through dual psychological pathways: employee satisfaction and perceived organizational support. We investigate two specific research questions: (1) How does leadership affect WFB directly and indirectly through employee satisfaction? (2) Through what mechanism does performance management influence WFB via perceived organizational support? Employing structural equation modeling with survey data from 469 employees across diverse industries, we test an integrated theoretical model grounded in boundary theory (Clark, 2000) and COR theory (Hobfoll, 1989).

This research makes theoretical and practical contributions to the work–family literature. Theoretically, we advance understanding of the complementary psychological mechanisms through which HRM practices influence WFB, demonstrating how employee satisfaction and perceived organizational support function as distinct resource-building pathways. Practically, our findings provide evidence-based guidance for organizations seeking to enhance work-family balance through targeted leadership development and performance management system redesign, thereby improving both employee well-being and organizational effectiveness.

## 2 Literature review

### 2.1 Theoretical foundations: integrating boundary theory and conservation of resources

The theoretical architecture undergirding work-family balance research rests predominantly on two complementary yet distinct frameworks: boundary theory and conservation of resources (COR) theory. These theoretical perspectives offer divergent but ultimately reconcilable explanations for how individuals navigate the complex interface between work and family domains, and how organizational practices influence this navigation process.

Boundary theory, initially articulated by Clark (2000) and substantially elaborated through the seminal work of Frone et al. (1992), conceptualizes work and family as distinct role domains separated by multidimensional boundaries. These boundaries possess three critical characteristics: permeability (the degree to which elements from one domain can enter another), flexibility (the extent to which boundaries can expand or contract), and blending (the degree to which elements from different domains are integrated). Clark's (2000) border theory specifically emphasizes that individuals function as border-crossers who make daily transitions between work and family domains, and the quality of these transitions depends fundamentally on boundary characteristics and the individual’s influence over them. Ashforth et al. (2000) extended this conceptualization by introducing the notion of micro role transitions, arguing that boundary management occurs not merely at macro-level domain shifts but through continuous micro-adjustments throughout the day. Their integration-segmentation continuum suggests that individuals vary in their preference for integrating versus segmenting work and family domains, and organizational practices can either support or constrain these preferences.

The theoretical power of boundary theory lies in its recognition that boundaries are socially constructed through negotiation with domain members and are influenced by organizational policies, supervisor behaviors, and workplace norms (Nippert-Eng, 1996). This social construction perspective implies that organizational practices—including leadership behaviors and performance management systems—function as boundary-shaping mechanisms. Supportive leadership may facilitate flexible boundary management by respecting non-work time and providing schedule autonomy, whereas rigid performance systems may create inflexible boundaries that intensify work–family conflict. However, boundary theory has been critiqued for insufficiently addressing why some individuals possess greater boundary management capacity than others and for inadequately specifying the psychological resources necessary for effective boundary negotiation (Allen et al., 2014).

Conservation of resources (COR) theory, developed by Hobfoll (1989) and subsequently refined through extensive empirical testing (Halbesleben et al., 2014; Hobfoll, 2001), addresses these limitations by providing a resource-centered framework. The theory posits that humans are fundamentally motivated to obtain, retain, and protect valued resources, which encompass object resources (e.g., housing, transportation), condition resources (e.g., employment, marriage), personal resources (e.g., self-efficacy, resilience), and energy resources (e.g., time, knowledge, money). COR theory’s two fundamental principles are particularly relevant for work-family research: primacy of resource loss (resource loss is disproportionately more salient than resource gain) and resource investment (individuals must invest resources to protect against resource loss, recover from losses, and gain new resources). These principles generate several corollaries, including the loss spiral corollary, which suggests that resource loss begets further loss, and the gain paradox corollary, which posits that resource gains become more salient in the context of resource loss.

Within the work-family context, COR theory suggests that organizational practices function as contextual resources [also termed resource caravans by Hobfoll (2001)] that either replenish or deplete employees’ personal resource reservoirs. Leadership behaviors providing emotional support, recognition, and autonomy represent resource gains that enhance employees’ capacity to manage competing role demands. Conversely, performance management systems emphasizing excessive monitoring or punitive evaluation may trigger resource loss through heightened job stress and reduced autonomy. The theory further predicts that resource-depleted individuals become defensive and focused on resource protection, potentially withdrawing effort from family domains to preserve remaining work-related resources, which in turn exacerbates work-family conflict (Brummelhuis and Bakker, 2012). The theoretical integration of boundary theory and COR theory thus provides a comprehensive framework: boundary theory explains the structural mechanisms of domain separation and integration, while COR theory explains the motivational and resource dynamics underlying boundary management capacity.

### 2.2 Empirical research: leadership, performance management, and psychological mechanisms

#### 2.2.1 Leadership behavior and work-family outcomes

Leadership behavior has emerged as a critical organizational antecedent of work-family balance, with accumulating evidence demonstrating that various leadership styles differentially influence employees’ capacity to navigate work-family boundaries. Li et al. (2017) conducted a comprehensive review synthesizing findings from 84 empirical studies, revealing that supportive leadership styles—characterized by empathy, flexibility, and genuine concern for employees’ non-work lives—consistently predict reduced work–family conflict and enhanced balance. Their meta-analytic findings indicated an average correlation of *r* = 0.21 between supportive leadership and work-family balance, with effect sizes varying substantially across cultural contexts and measurement approaches.

Transformational leadership, conceptualized as leadership that inspires followers through idealized influence, inspirational motivation, intellectual stimulation, and individualized consideration (Bass, 1985), has demonstrated particularly robust associations with work-family outcomes. Transformational leaders may enhance work-family balance through multiple pathways: reducing role ambiguity and conflict through clear communication, providing emotional support during stressful periods, and modeling healthy work-family integration behaviors. However, Huang et al. (2021) caution that transformational leadership’s emphasis on high performance expectations and emotional engagement may paradoxically intensify work demands, potentially offsetting its positive effects. Their longitudinal study revealed a curvilinear relationship, with moderate levels of transformational leadership optimally supporting work-family balance while extreme levels generated excessive demands.

Servant leadership, characterized by prioritizing follower development and well-being over organizational objectives, has received increasing empirical attention in work-family research. Park et al. (2023) found that servant leadership reduces work–family conflict through a dual-mediation pathway involving gratitude and work engagement. Their structural equation modeling revealed that servant leaders’ authentic concern for employees’ holistic well-being generates felt gratitude, which subsequently enhances work engagement and provides psychological resources for managing family responsibilities. Stollberger et al. (2019) extended these findings by demonstrating trickle-down effects, whereby servant leadership shapes not only direct reports’ work-family balance but also influences their own leadership behaviors toward subordinates, creating cascading effects throughout organizational hierarchies. Their three-wave longitudinal design provided compelling evidence that leadership effects on work-family outcomes operate through both direct and indirect pathways.

Leader-member exchange (LMX) quality has emerged as a particularly consequential relational factor. High-quality LMX relationships, characterized by mutual trust, respect, and obligation, provide employees with greater autonomy, informal flexibility, and psychological safety to negotiate work-family boundaries (Wayne et al., 2020). Golden and Fromen (2011) demonstrated that LMX quality moderates the relationship between work demands and work–family conflict, with high LMX buffering against the negative effects of job demands. However, Bagger and Li (2014) identified a potential dark side: employees in high-quality LMX relationships may feel obligated to reciprocate through intensified work effort and extended availability, potentially undermining work-family balance. This reciprocity obligation may be particularly salient in collectivistic cultures where interpersonal harmony and obligation fulfillment are paramount values.

#### 2.2.2 Performance management systems and work-family Interface

Despite the pervasive influence of performance management systems on employee work experiences, their role in shaping work-family outcomes has received comparatively limited systematic attention. Performance management encompasses multiple interconnected processes, including goal-setting, performance monitoring, feedback provision, evaluation, and reward allocation (DeNisi and Murphy, 2017). Each component potentially influences work-family balance through distinct mechanisms, yet most research examines performance management holistically rather than disaggregating its constituent elements.

Na-Nan et al. (2020) argue that performance management systems in contemporary organizations face fundamental tensions between productivity imperatives and employee quality of life considerations. Their qualitative study of manufacturing firms revealed that employees perceive performance systems emphasizing quantitative metrics and short-term results as incompatible with work-family balance, generating pressure to prioritize work over family obligations. Conversely, performance systems incorporating developmental feedback, recognizing non-work contributions, and providing flexibility in goal achievement timelines were perceived as supporting work-family integration. These findings align with self-determination theory (Deci and Ryan, 2000), suggesting that performance systems satisfying basic psychological needs for autonomy, competence, and relatedness enhance rather than undermine well-being outcomes.

Goal-setting characteristics exert particularly substantial influence on work-family dynamics. Locke and Latham's (2002) goal-setting theory posits that specific, challenging goals enhance performance but may simultaneously intensify work demands and reduce flexibility. Maertz and Boyar (2011) found that when performance goals are perceived as excessively difficult or conflicting, employees experience heightened work–family conflict through increased work hours and psychological preoccupation with work during non-work time. However, goal-setting effects appear contingent on implementation characteristics. When employees participate in goal-setting processes and perceive goals as personally meaningful and aligned with broader life objectives, performance goals may actually enhance work-family balance by providing clarity and reducing uncertainty (Baral and Bhargava, 2010).

Performance feedback represents another critical component with nuanced effects. Vidè et al. (2023) investigated how performance appraisal purposes (developmental versus administrative) differentially influence employee attitudes. Their study revealed that when employees perceived performance evaluations as primarily developmental—focused on skill enhancement and career growth—they reported higher job engagement and lower work–family conflict. Conversely, evaluations perceived as purely administrative decision-making tools generated anxiety and defensive responses that spilled over into family domains. The frequency and quality of feedback also matter considerably. Bankins et al. (2024) examined the integration of artificial intelligence in performance management, finding that algorithm-based continuous feedback systems were perceived as invasive and stress-inducing, potentially exacerbating work–family conflict through the erosion of psychological boundaries between work and non-work time.

#### 2.2.3 Psychological mechanisms: employee satisfaction and perceived organizational support

Employee satisfaction and perceived organizational support have been identified as potentially crucial psychological mechanisms linking organizational practices to work-family outcomes, yet their specific mediating roles remain inadequately theorized and empirically underexplored. These constructs, while conceptually related, represent distinct psychological phenomena with different theoretical foundations and potentially divergent mediating pathways.

Employee satisfaction, conceptualized as a positive affective state resulting from job appraisal (Locke, 1976), represents an immediate emotional response to work experiences. From a COR perspective, job satisfaction functions as a psychological resource that enhances individuals’ capacity to navigate work-family boundaries effectively. Bellmann and Hübler (2021) demonstrated that job satisfaction mediates the relationship between flexible work arrangements and work-family balance, suggesting that satisfaction operates as a proximal psychological state connecting organizational practices to work-family outcomes. Their longitudinal analysis revealed that satisfaction’s mediating effect persisted even after controlling for objective work characteristics, indicating that subjective appraisals matter independently of objective conditions.

Wan et al. (2022) provided compelling evidence for reciprocal relationships between job satisfaction and work-family balance. Their longitudinal study of nurses demonstrated that job satisfaction at Time 1 predicted work-family balance at Time 2, which subsequently predicted job satisfaction at Time 3, suggesting positive feedback loops. These reciprocal dynamics align with the gain spiral corollary of COR theory, whereby initial resource gains facilitate subsequent gains through enhanced resource investment capacity. However, Wan et al.’s findings also revealed that the satisfaction-to-balance pathway was stronger than the balance-to-satisfaction pathway, suggesting asymmetric causality that warrants further investigation.

Perceived organizational support (POS), defined as employees’ global beliefs concerning the extent to which organizations value their contributions and care about their well-being (Eisenberger et al., 1986), operates through a somewhat distinct mechanism grounded in organizational support theory. This theory posits that POS develops through employees’ attributions about organizational actions, with discretionary favorable treatment and support during difficult times generating particularly strong POS. Unlike job satisfaction, which reflects immediate affective responses, POS represents more stable cognitive beliefs about the employment relationship that shape expectations about how organizations will behave in future situations (Kurtessis et al., 2017).

Kurtessis et al.'s (2017) comprehensive meta-analysis synthesizing 558 effect sizes revealed that POS demonstrates consistent associations with work-family outcomes (*ρ* = 0.34), but these relationships are often indirect rather than direct. POS influences work-family balance primarily by shaping how employees interpret organizational demands and their felt obligations to reciprocate organizational support. High POS may buffer against work–family conflict by providing psychological security that the organization will accommodate family needs when necessary, thereby reducing anticipatory anxiety about potential conflicts. Furthermore, POS may influence boundary management preferences; employees perceiving high organizational support may feel more comfortable establishing boundaries and declining excessive work demands without fearing negative career consequences.

Despite conceptual distinctions, employee satisfaction and POS have rarely been examined simultaneously as potentially complementary mediating mechanisms. Existing research tends to investigate these constructs in isolation, precluding assessment of their relative importance or examination of whether they mediate relationships with different organizational antecedents. Theoretically, we might expect differential mediation patterns: leadership behaviors, operating through proximal interpersonal interactions, may influence work-family balance primarily through job satisfaction, whereas performance management systems, signaling organizational values and priorities, may operate primarily through POS. However, this differential mediation hypothesis remains empirically untested.

### 2.3 Critical gaps and theoretical limitations

Despite substantial empirical progress, the work–family literature remains characterized by several critical gaps and theoretical limitations that constrain both scholarly understanding and practical application. These limitations span conceptual, methodological, and theoretical domains, collectively hindering development of comprehensive models capable of guiding organizational interventions.

First, leadership and performance management have predominantly been examined independently, with minimal research exploring their potential synergistic, compensatory, or interactive effects. This fragmented approach reflects broader disciplinary divisions, with leadership research typically originating from organizational behavior traditions emphasizing interpersonal dynamics, while performance management research emerges from strategic HRM traditions emphasizing systems and practices. However, organizations do not implement these practices in isolation; they function as integrated HRM systems whose combined influence may differ substantially from their isolated effects (Daraba et al., 2021). For instance, supportive leadership might compensate for rigid performance systems by providing informal flexibility, or alternatively, rigid performance systems might undermine supportive leadership by constraining leaders’ discretionary capacity. These interactive dynamics remain empirically uncharted territory.

Second, while employee satisfaction and perceived organizational support are conceptually relevant psychological mechanisms, their specific mediating roles connecting HRM practices to work-family outcomes remain empirically underexplored. Most existing research treats these constructs as outcomes rather than mediating mechanisms, or examines them individually rather than comparing their relative importance within integrated models (Isa and Indrayati, 2023). This limitation is particularly consequential because understanding mediation pathways has direct practical implications: if leadership influences work-family balance primarily through satisfaction while performance management operates through POS, interventions should target different psychological mechanisms depending on the organizational practice being modified. Furthermore, the literature lacks systematic investigation of whether these mediating mechanisms operate equivalently across cultural contexts or differ based on cultural values regarding authority, collectivism, and work centrality.

Third, insufficient theoretical integration characterizes this literature. Boundary theory and COR theory are rarely synthesized within unified frameworks despite their complementarity. Boundary theory excels at explaining structural mechanisms of domain separation and the social construction of boundaries but provides limited insight into individual differences in boundary management capacity or the motivational dynamics underlying boundary negotiations. COR theory addresses these limitations by specifying resource dynamics but offers less precise predictions about how specific organizational practices shape particular boundary characteristics. Integrative frameworks combining these perspectives remain underdeveloped, limiting theoretical advancement (Allen et al., 2014). This theoretical fragmentation manifests in empirical research through atheoretical variable selection, inconsistent construct measurement, and limited specification of mediating mechanisms.

### 2.4 Research positioning, questions, and hypothesized model

This study addresses the identified gaps through an integrative examination of how leadership behavior and performance management practices jointly influence work-family balance via dual psychological pathways. We advance a differential mediation model proposing that these HRM practices operate through distinct psychological mechanisms reflecting their different natures and proximity to employees’ daily experiences. Specifically, we hypothesize that leadership behavior—operating through proximal interpersonal interactions and shaping immediate work experiences—influences work-family balance both directly and indirectly through employee satisfaction. In contrast, we propose that performance management systems—functioning as organizational structures signaling institutional values and priorities—influence work-family balance primarily through perceived organizational support rather than immediate affective states.

This differential mediation framework is theoretically grounded in the integrated boundary theory and COR perspective. Leadership behaviors influence employees’ immediate boundary management capacity by providing social support, flexibility, and emotional resources that facilitate daily transitions between work and family domains. These immediate experiences generate affective responses (satisfaction) that function as psychological resources enhancing boundary management capacity. Performance management systems, operating at a more distal level, shape employees’ perceptions of organizational values and the extent to which the organization supports their holistic well-being (POS). These perceptions influence work-family balance by shaping employees’ attributions about whether organizational demands are legitimate and whether they can safely establish boundaries without career penalties.

By testing this integrated model using structural equation modeling with data from 469 employees across diverse industries, we aim to clarify the complementary yet distinct mechanisms through which organizations can enhance employee work-family balance. This approach offers both theoretical and practical advantages. Theoretically, it advances understanding of how organizational practices simultaneously operate through affective and cognitive pathways to shape work-family outcomes. Practically, it provides evidence-based guidance regarding which psychological mechanisms should be targeted when modifying specific organizational practices to improve work-family balance.

## 3 Research methodology

### 3.1 Research design

This study employed a quantitative research design to examine the relationships between human resource management practices (specifically leadership behavior and performance management) and work-family balance, with particular attention to the mediating roles of employee satisfaction and perceived organizational support. A cross-sectional survey methodology was adopted as the primary data collection approach, supplemented by semi-structured interviews to provide contextual depth and interpretive insights (Creswell and Creswell, 2018).

**Figure 1:**
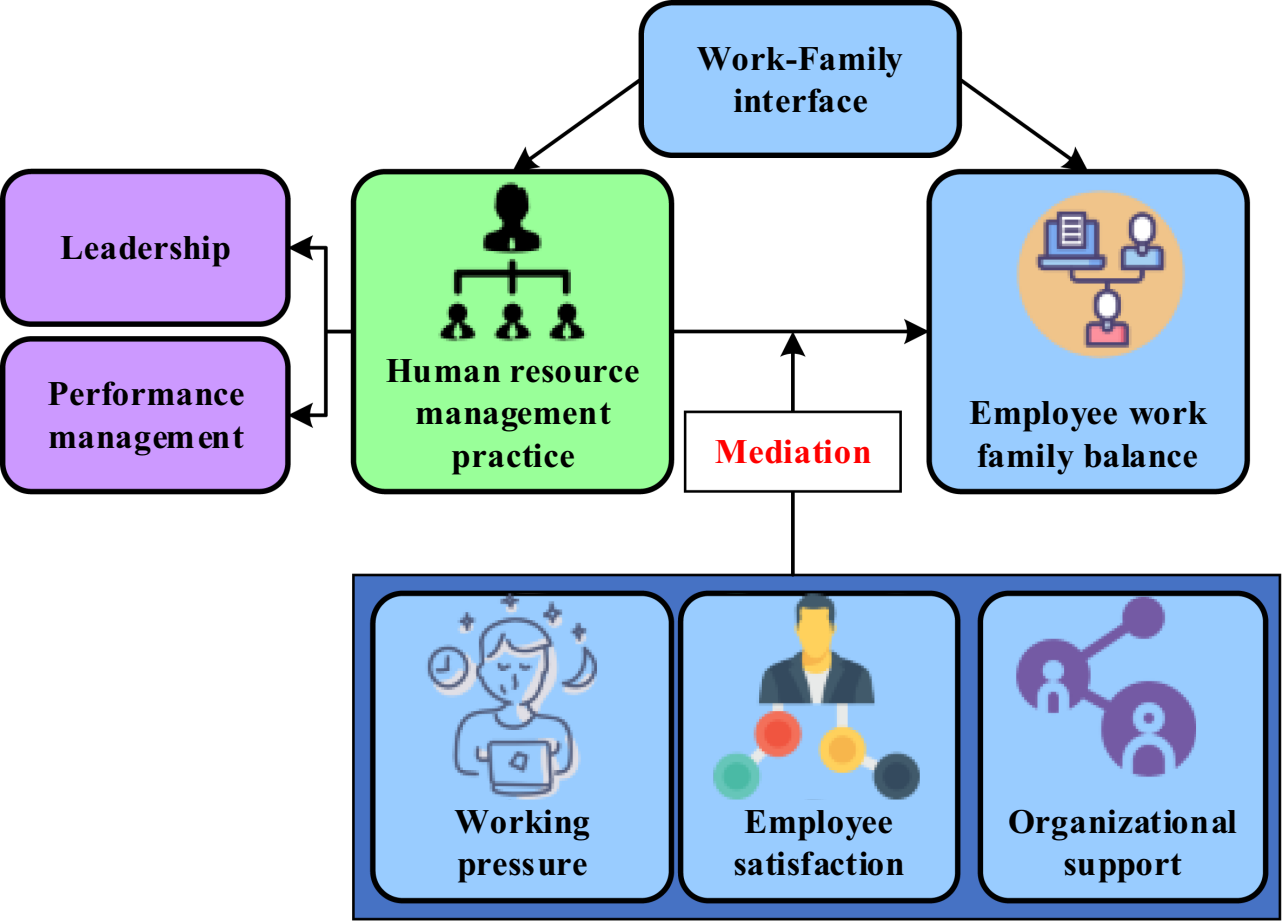
Schematic diagram of the research framework.

The theoretical framework guiding this investigation was grounded in boundary theory (Clark, 2000) and conservation of resources theory (Hobfoll, 1989), as articulated in the literature review. The research model posited three mediating pathways through which HRM practices influence work-family balance: (1) the effect of leadership behavior on work-family balance mediated by employee satisfaction, (2) the effect of performance management on work-family balance mediated by perceived organizational support, and (3) the effect of both HRM practices on work-family balance mediated by work pressure. The conceptual framework illustrating these hypothesized relationships is presented in Figure 1.

A cross-industry sampling strategy was implemented to ensure sample representativeness and enhance the generalizability of findings (Podsakoff et al., 2012). Organizations were recruited from diverse sectors including manufacturing, services, information technology, and finance. This multi-industry approach enabled examination of the proposed relationships across varied organizational contexts while controlling for industry-specific effects. Enterprise characteristics such as organizational size, geographic location, and ownership structure were recorded to facilitate subsequent subgroup analyses and assessment of contextual moderators.

### 3.2 Data collection

#### 3.2.1 Quantitative survey sample

**Figure 2:**
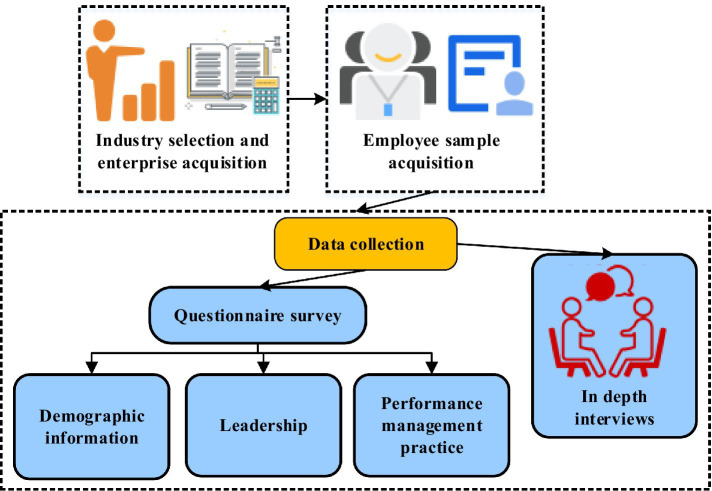
The process of sample selection and data collection.

A multi-stage sampling procedure was employed to recruit organizational participants and individual respondents. The sampling process is illustrated in Figure 2. In the initial stage, target industries and candidate organizations were identified through professional networking platforms (LinkedIn) and domestic industry association databases. A purposive sampling approach was utilized to select organizations meeting predefined eligibility criteria: (1) minimum of 100 employees, (2) established HR management systems, and (3) willingness to participate in academic research. Organizations were contacted through official channels, with research objectives and procedures explained to human resources departments.

Following organizational consent, employee recruitment was conducted through internal communication channels including organizational email systems, notice boards, and corporate social networks. Participation was voluntary, and respondents were assured of complete anonymity and confidentiality. To enhance response rates and data quality, the research purpose was clearly communicated, emphasizing potential contributions to improved work-family balance policies. No incentives were provided to maintain response authenticity.

Survey data were collected through two primary methods: electronic questionnaires and paper-based surveys, depending on organizational preferences and technological infrastructure. Online surveys were administered via established platforms (SurveyMonkey and Tencent Questionnaires), while paper surveys were distributed through HR departments with sealed return envelopes to ensure confidentiality. A pilot test involving 30 employees was conducted prior to full-scale data collection to assess questionnaire comprehensibility, identify ambiguous items, and evaluate completion time. Based on pilot feedback, minor wording adjustments were implemented to enhance clarity.

A total of 500 questionnaires were distributed across participating organizations. After eliminating incomplete responses and surveys demonstrating obvious response patterns (e.g., straight-lining), 469 valid questionnaires were retained, yielding an effective response rate of 93.8%. This response rate exceeded typical benchmarks for organizational surveys (Baruch and Holtom, 2008) and provided sufficient statistical power for the planned analyses.

#### 3.2.2 Qualitative interview sample

To complement quantitative findings and provide deeper contextual understanding, semi-structured interviews were conducted with a purposefully selected subsample of survey participants. Interview participant selection followed rigorous criteria designed to maximize data richness and diversity: (1) minimum 24 months of continuous organizational tenure to ensure adequate experience with organizational practices, (2) significant family responsibilities operationalized as marriage/committed partnership, dependent children under 18 years, or eldercare responsibilities, and (3) voluntary consent with availability for 45–60 min interviews.

A maximum variation sampling strategy was implemented following established qualitative research protocols (Patton, 2015). The final interview sample (*N* = 30) achieved balanced gender representation (47% male, 53% female) and included participants across three hierarchical levels (entry, mid-level, senior) and four industry sectors (manufacturing, services, IT, finance). Interviews were conducted via remote video conferencing platforms (Zoom, WeChat Video) to accommodate geographic dispersion and scheduling constraints. All interviews were audio-recorded with participant consent and transcribed verbatim for analysis.

Data saturation was systematically assessed throughout the interview process (Guest et al., 2006). No substantial new themes emerged after the 25th interview, and five additional interviews were conducted to confirm saturation. Interview protocols were semi-structured, allowing flexibility to explore emergent themes while ensuring systematic coverage of key research topics including perceptions of leadership support, performance management experiences, and work-family boundary management strategies.

### 3.3 Measurement

All constructs were measured using established scales from prior research, adapted where necessary to fit the study context. All items employed five-point Likert scales ranging from 1 (strongly disagree) to 5 (strongly agree) to maintain measurement consistency and facilitate statistical comparison. Scale selection prioritized instruments with demonstrated reliability and validity in organizational research contexts.

*Leadership behavior*: Leadership behavior was assessed using a five-item scale adapted from Bass and Avolio (1997), focusing on supportive and transformational leadership dimensions relevant to work-family outcomes. Items evaluated leader behaviors including encouragement of employee participation in decision-making, provision of clear goals and expectations, timely performance feedback, demonstration of concern for employee well-being, and support for work-life integration. Sample items included “My supervisor encourages me to participate in important decisions” and “My supervisor shows concern for my personal and family needs.”

*Performance management practices*: Performance management was measured using a five-item scale assessing perceived fairness, transparency, and developmental orientation of organizational performance systems (DeNisi and Pritchard, 2006). Items addressed frequency of performance evaluations, clarity of performance criteria, fairness of evaluation processes, quality and timeliness of performance feedback, and alignment between performance expectations and resource provision. Representative items included “Performance evaluations in my organization are conducted fairly” and “I receive constructive feedback that helps me improve my performance.”

*Employee satisfaction*: Job satisfaction was assessed using the Job Descriptive Index (JDI; Smith et al., 1969), a widely validated multidimensional measure. The scale comprised 15 items across five facets: work content satisfaction (3 items), compensation satisfaction (3 items), promotion opportunities (3 items), supervision satisfaction (3 items), and coworker relationships (3 items). This multifaceted approach enabled assessment of overall job satisfaction while permitting examination of dimension-specific effects if warranted by analyses.

*Perceived organizational support*: POS was measured using an eight-item scale adapted from Eisenberger et al. (1986), the seminal measure in organizational support research. Items assessed employees’ perceptions of organizational valuation of their contributions, concern for their well-being, consideration of their goals and values, and willingness to provide assistance when needed. Sample items included “My organization values my contributions to its well-being” and “My organization shows concern for my welfare.”

*Work pressure*: Work pressure was evaluated using a 12-item Work Pressure Scale measuring stress arising from job demands, time pressures, and role conflicts. The scale assessed multiple pressure dimensions including workload intensity, time constraints, competing demands, resource inadequacy, and role ambiguity. Higher scores indicated greater perceived work pressure and stress.

*Work-family balance*: WFB was measured using the Work–Family Conflict Scale and Family–Work Conflict Scale developed by Netemeyer et al. (1996), each comprising 10 items. These bidirectional measures assessed the extent to which work interferes with family responsibilities (work-to-family conflict) and family interferes with work responsibilities (family-to-work conflict). The 20-item composite provided comprehensive assessment of work-family interface difficulties. Sample items included “The demands of my work interfere with my home and family life” and “The demands of my family interfere with my work responsibilities.” Scores were reverse-coded such that higher values indicated greater work-family balance (lower conflict).

*Demographic variables*: Standard demographic information was collected including age, gender, organizational tenure, job level, industry sector, marital status, and number of dependent children. These variables served as control variables in statistical analyses and enabled assessment of sample representativeness.

Scale reliability and validity were assessed through pilot testing and confirmed in the main study. Cronbach’s alpha coefficients for all scales exceeded the conventional threshold of 0.70, indicating acceptable internal consistency. Confirmatory factor analysis was conducted to assess construct validity, with results demonstrating adequate model fit and discriminant validity among constructs. The complete reliability and validity assessment results are presented in Table 1.

**Table 1:** Results of reliability and validity testing.

Questionnaire content	Cronbach’s *α*	Content validity	Construct validity
Leadership	0.85	High	Meets expectations
PM practice	0.82	High	Meets expectations
ES	0.88	High	Meets expectations
POS	0.90	High	Meets expectations
Work pressure	0.76	High	Meets expectations
WFB	0.89	High	Meets expectations

### 3.4 Data analysis

**Figure 3:**
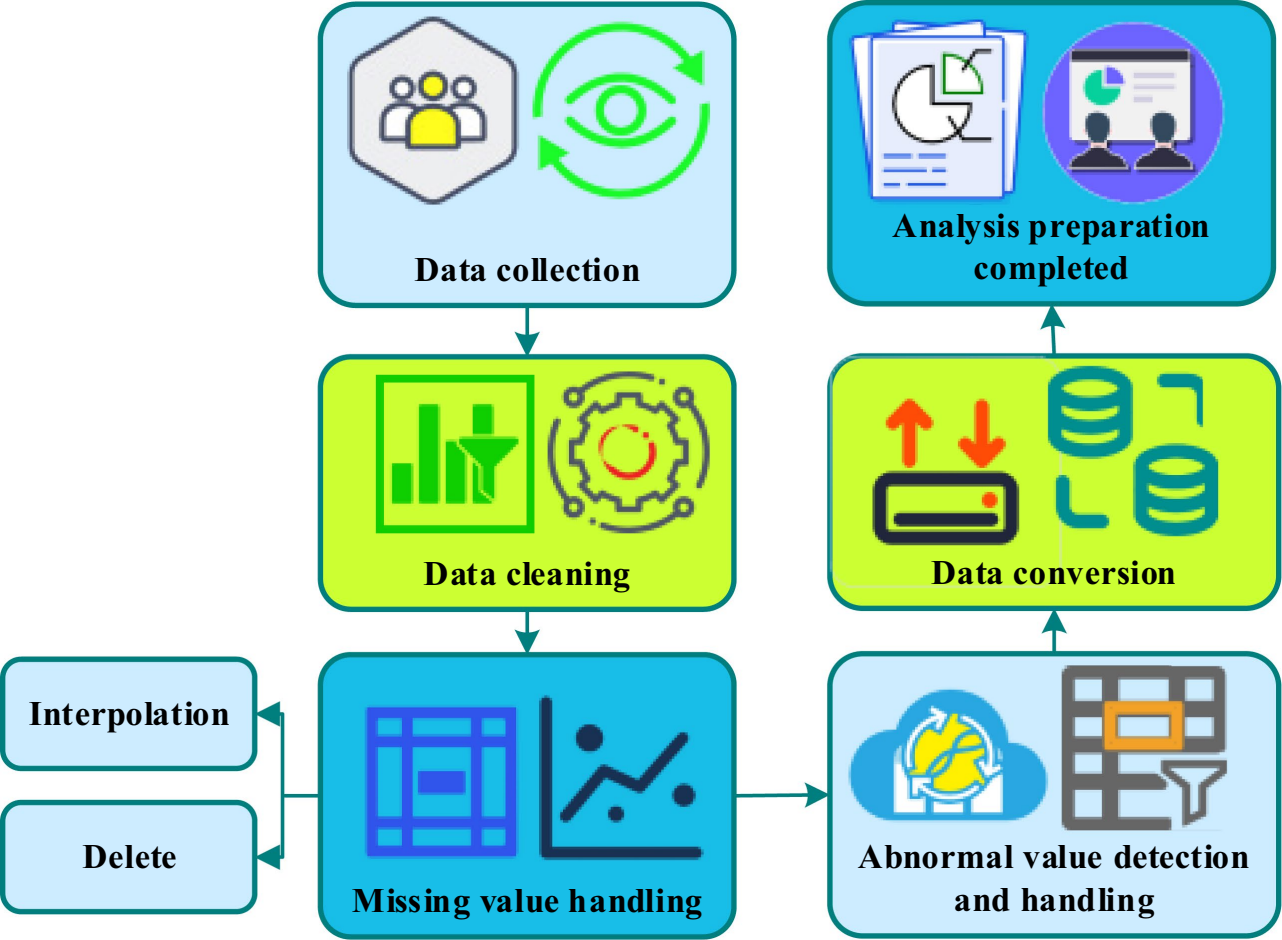
The flow of data preprocessing.

Data analysis proceeded through sequential stages, beginning with data screening and cleaning, followed by preliminary descriptive and correlational analyses, and culminating in structural equation modeling to test hypothesized relationships. The data preprocessing workflow is illustrated in Figure 3.

*Data screening and cleaning*: Initial data screening examined missing values, outliers, and response patterns indicating careless responding. Missing values were assessed using Little’s MCAR test to determine whether data were missing completely at random. Cases with more than 10% missing values were excluded from analysis. For cases with minimal missing data, multiple imputation procedures were employed (Schafer and Graham, 2002). Univariate and multivariate outliers were identified through examination of standardized scores (|z| > 3.29) and Mahalanobis distance values. Outliers were evaluated for potential data entry errors, and legitimate extreme values were retained to preserve sample representativeness. Data distributions were examined for normality violations through inspection of skewness and kurtosis statistics, with transformations applied where necessary to meet statistical assumptions.

*Descriptive and correlational analyses*: Descriptive statistics including means, standard deviations, and frequency distributions were calculated for all study variables to characterize the sample and assess variable distributions. Bivariate correlations among all study variables were examined using Pearson correlation coefficients to provide preliminary evidence regarding hypothesized relationships and identify potential multicollinearity concerns. Correlation matrices were inspected for correlations exceeding 0.80, which might indicate problematic multicollinearity (Grewal et al., 2004).

*Structural equation modeling*: The primary analytic technique employed was structural equation modeling (SEM) using AMOS 24.0 software. SEM was selected for its capacity to simultaneously test measurement models and structural relationships while accounting for measurement error (Kline, 2016). The analysis proceeded through a two-step approach recommended by Anderson and Gerbing (1988). First, confirmatory factor analysis (CFA) was conducted to assess the measurement model, examining whether observed indicators adequately represented latent constructs and whether constructs demonstrated discriminant validity. Model fit was evaluated using multiple indices including chi-square statistic (χ^2^), comparative fit index (CFI), Tucker-Lewis index (TLI), root mean square error of approximation (RMSEA), and standardized root mean square residual (SRMR). Acceptable fit was indicated by CFI and TLI values exceeding 0.90, RMSEA values below 0.08, and SRMR values below 0.08 (Hu and Bentler, 1999).

Following confirmation of adequate measurement model fit, the structural model was estimated to test hypothesized direct and indirect effects. Mediation hypotheses were examined through analysis of indirect effects using bootstrapping procedures with 5,000 resamples to generate bias-corrected confidence intervals (Preacher and Hayes, 2008). Indirect effects were considered statistically significant when 95% confidence intervals excluded zero. To assess relative magnitudes of mediation effects, proportions of mediated effects were calculated by dividing indirect effects by total effects.

*Supplementary analyses*: Several supplementary analyses were conducted to provide additional insights. First, hierarchical regression analysis was performed to examine the incremental variance explained by HRM practices beyond demographic control variables. Second, moderation analyses were conducted to assess whether relationships varied significantly across demographic subgroups (gender, organizational tenure, job level). Third, qualitative interview data were analyzed using thematic analysis procedures (Braun and Clarke, 2006), with themes inductively identified and subsequently mapped onto quantitative findings to provide interpretive depth. Inter-rater reliability for qualitative coding was established through independent coding of 30% of transcripts by two researchers, achieving satisfactory agreement (*κ* = 0.82).

### 3.5 Data quality control and common method bias assessment

Given the reliance on self-reported survey data collected from a single source, rigorous procedures were implemented to minimize common method bias (CMB) and ensure data quality. Both procedural remedies during data collection and statistical controls during data analysis were employed following recommendations by Podsakoff et al. (2003, 2012).

#### 3.5.1 Procedural remedies

*Temporal separation*: A two-wave data collection design was implemented to introduce temporal separation between measurement of predictor and criterion variables. At Time 1 (baseline), independent variables (leadership behavior, performance management practices, work pressure) and demographic characteristics were measured. At Time 2 (4 weeks later), mediating variables (employee satisfaction, perceived organizational support) and the outcome variable (work-family balance) were assessed. This temporal separation reduces same-source bias by preventing respondents from using their earlier responses to guide later responses, thereby diminishing consistency artifacts and implicit theories (Podsakoff et al., 2003). The four-week interval was selected to balance practical feasibility against the benefits of temporal separation, following recommendations that intervals of 2–6 weeks provide meaningful separation while minimizing attrition (Doty and Glick, 1998).

*Anonymity and confidentiality assurance*: Participants were explicitly informed that their responses would be completely anonymous and that no individual-level results would be shared with employers. To facilitate matching of Time 1 and Time 2 responses while preserving anonymity, each participant generated a unique self-created identification code based on predetermined rules (e.g., mother’s birth month, last two digits of phone number). This approach has been demonstrated to reduce evaluation apprehension and social desirability bias (Podsakoff et al., 2003). Survey instructions emphasized that there were no right or wrong answers and encouraged honest responding.

*Item randomization*: To minimize consistency effects and reduce respondents’ ability to discern hypothesized relationships, items measuring different constructs were randomly intermixed rather than blocked by construct. This randomization was implemented within survey sections to maintain logical flow while disrupting potential heuristic processing patterns.

*Scale format variation*: Different response scale formats were employed across constructs to reduce method biases associated with consistent scale use. While all scales maintained five-point ranges for comparability, item stems and anchor labels varied. For example, leadership behavior items used frequency anchors (“never” to “always”), while work-family balance items used agreement anchors (“strongly disagree” to “strongly agree”). This variation makes it more difficult for respondents to maintain consistent response patterns across all items (Podsakoff et al., 2003).

#### 3.5.2 Statistical controls and assessment

*Harman’s single-factor test*: As a preliminary assessment of CMB severity, Harman’s single-factor test was conducted through exploratory factor analysis with all items loading onto a single factor (Podsakoff and Organ, 1986). Results indicated that the single factor explained only 28.7% of total variance, well below the 50% threshold typically indicating substantial CMB concerns. While this test has been criticized for limited sensitivity, it provides initial evidence that CMB is not pervasive in the data.

*Confirmatory factor analysis comparison*: A more rigorous CMB assessment compared the fit of the hypothesized measurement model (with distinct factors for each construct) against a single-factor model wherein all items loaded onto one common method factor. The hypothesized model (χ^2^/df = 2.45, RMSEA = 0.05, CFI = 0.96, TLI = 0.95) demonstrated significantly superior fit compared to the single-factor model (χ^2^/df = 8.73, RMSEA = 0.14, CFI = 0.67, TLI = 0.64), Δχ^2^(15) = 2,847.3, *p* < 0.001. This substantial improvement in fit provides compelling evidence that CMB does not account for the covariation among measures (Williams et al., 1989).

*Common latent factor test*: As an additional safeguard, the common latent factor (CLF) technique was implemented by including an unmeasured latent method factor in the structural model with all observed indicators loading on both their substantive constructs and the CLF (Podsakoff et al., 2003). Comparison of standardized regression weights with and without the CLF revealed minimal differences (Δβ < 0.05 for all paths), suggesting that common method variance does not substantially bias the structural parameter estimates.

Collectively, these procedural remedies and statistical controls provide reasonable assurance that common method bias does not pose a serious threat to the validity of the study findings. However, the inherent limitations of self-report data are acknowledged. Future research would benefit from incorporating multi-source ratings, including supervisor assessments of leadership behavior and employee performance, peer ratings of work-family balance, and objective organizational records where feasible.

The methodological procedures outlined in this section were designed to ensure data quality, minimize bias, and enable rigorous testing of the proposed theoretical model. Particular attention was devoted to construct measurement validity, common method bias mitigation, and appropriate statistical techniques for examining complex mediation relationships.

## 4 Results and discussion

### 4.1 Sample characteristics and descriptive statistics

Questionnaire data were systematically organized to examine the distribution of samples based on key demographic characteristics. Among the 469 valid respondents, there are 250 males (53%) and 219 females (47%), indicating a nearly balanced gender ratio. Such gender balance is particularly important for examining work-family balance issues, as prior research has demonstrated that men and women often experience different types and intensities of work–family conflict (Bellmann and Hübler, 2021). Balanced representation enables examination of gender-related patterns in how HRM practices influence work-family outcomes.

**Figure 4:**
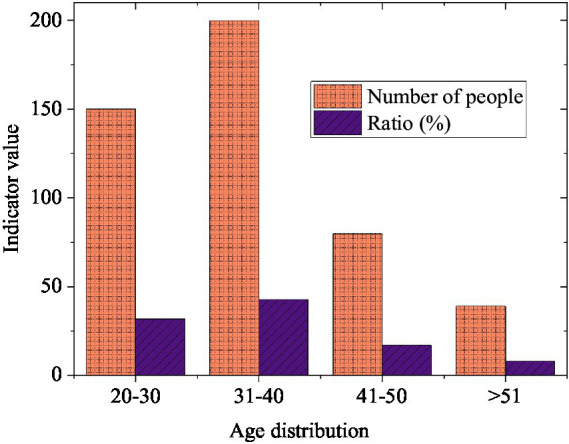
Age distribution.

Figure 4 presents the age distribution of the sample. A wide-ranging age distribution indicates that the research sample covers employees in diverse stages of life. Early studies suggest that conflicts between work and family roles may manifest various trends among employees of different age groups, with younger employees often facing challenges related to establishing career trajectories while older employees may contend with elder care responsibilities (Williams and McCombs, 2023). Thus, this age diversity helps to comprehensively understand the varied requirements for work-family balance across different life stages.

**Figure 5:**
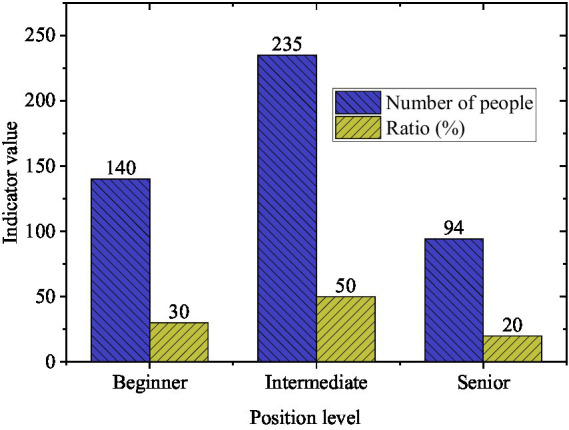
Distribution of position levels.

Regarding organizational position level, Figure 5 illustrates that mid-level employees account for the highest proportion of the sample. This concentration is theoretically meaningful, as mid-level employees typically occupy what has been termed the “sandwich” position—simultaneously facing upward accountability to senior management and downward responsibility for subordinates, while also managing increasing family obligations. This demographic profile aligns well with life-course theories suggesting that work–family conflict peaks during these mid-career years when both professional and family demands are at their highest (Petitta et al., 2024).

**Figure 6:**
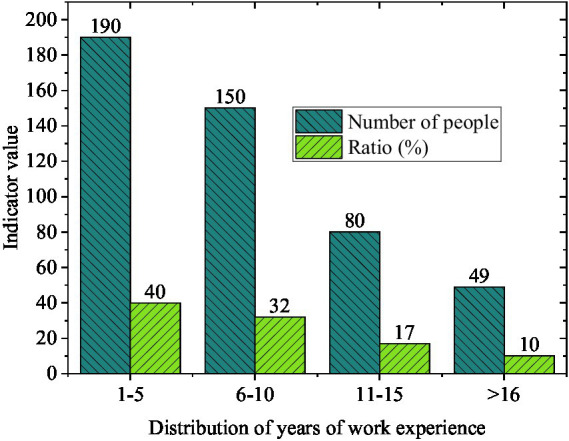
Distribution of years of work experience.

Figure 6 displays the distribution of years of work experience. Most employees have 1–10 years of work experience, indicating that the sample primarily comprises individuals at crucial stages of career development. During this period, employees often experience increasing work pressure alongside growing family responsibilities, such as marriage, childbirth, and childcare, which collectively pose higher demands on work-family balance (Wan et al., 2022). However, this concentration may also limit the generalizability of findings to entry-level employees or senior executives who may experience different work-family dynamics.

**Figure 7:**
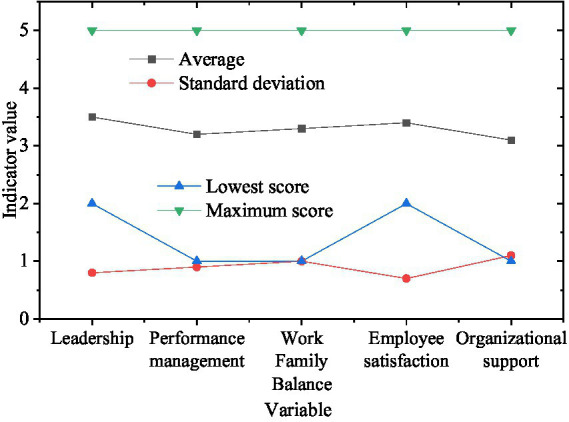
Descriptive statistics of HRMP and WFB.

Figure 7 provides detailed descriptive statistics for HRM practices and employees’ perception of WFB. Average scores of leadership and employee satisfaction (ES) are relatively high, at 3.5 and 3.4 respectively, indicating that employees generally perceive good leadership as contributing to enhanced ES. This finding provides preliminary support for the hypothesized positive relationship between supportive leadership and employee psychological outcomes. Leadership, conceptualized as an organizational resource within the Conservation of Resources (COR) framework, may help employees better balance work and family life by reducing uncertainty and conflicts at work (Hobfoll, 1989). Supportive leaders provide emotional resources through empathetic communication, instrumental resources through workload management, and informational resources through clear expectations—all of which facilitate employees’ capacity to manage work-family boundaries effectively. Furthermore, performance management (PM) and perceived organizational support (POS) have lower average scores of 3.2 and 3.1, illustrating possible deficiencies in these areas that warrant organizational attention. Relatively lower PM scores may indicate that employees perceive current performance evaluation systems as inadequately supportive of their developmental needs or insufficiently flexible to accommodate family responsibilities. Similarly, lower POS scores suggest opportunities for organizations to enhance family-friendly policies and demonstrate greater concern for employees’ non-work lives. These findings suggest that organizations may need to pay more attention to individual differences and flexibility in PM systems, and enhance POS through family-friendly policies and flexible work arrangements.

It is important to acknowledge that the demographic composition of our sample, while providing valuable insights, also presents certain limitations. Predominance of mid-level employees with 1–10 years of work experience offers a particularly valuable window into the “sandwich generation” phenomenon—employees simultaneously facing increasing career responsibilities and growing family obligations. However, entry-level employees, who may face different challenges such as establishing career identity while managing early family formation, are underrepresented. Similarly, senior executives, who typically possess greater autonomy and decision-making authority to manage work-family boundaries, may experience different dynamics than our sample reveals. Future research should ensure more balanced representation across career stages to capture the full spectrum of work-family balance experiences.

### 4.2 Measurement validation

Exploratory factor analysis (EFA) was conducted to assess the factor structure of the measurement instruments. Results are exhibited in Table 2.

**Table 2:** The EFA results.

Factor	Leadership	PM	ES	POS	Work pressure	WFB
Factor 1 (Leadership)	0.82			0.75		
Factor 2 (PM)		0.80			0.76	
Factor 3 (ES)			0.85			0.70
Factor 4 (POS)	0.68		0.77	0.83		
Factor 5 (work pressure)		0.79			0.88	
Factor 6 (WFB)			0.72		0.65	0.90

Items with factor loadings<0.4 are not shown.

EFA results in Table 2 demonstrate that the composition of each factor is consistent with theoretical expectations, and each variable exhibits high factor loadings on the corresponding factors. This indicates that the structure of the questionnaire effectively reflects the preset dimensions and confirms the appropriateness of treating these constructs as distinct yet related aspects of the work-family interface. Clear differentiation of variables, such as leadership and PM, validates the structural validity of the measurement tools and supports the theoretical distinction between different types of HRM practices. Cronbach’s *α* values range from 0.85 to 0.91, demonstrating good internal consistency among the measurement dimensions and exceeding the commonly accepted threshold of 0.70 for reliable measurement. Composite reliability of all dimensions exceeds the recommended value of 0.7, and the average variance extracted (AVE) surpasses the critical value of 0.5, thus confirming the questionnaire’s reliability and construct validity according to established psychometric criteria. Discriminant validity was further established by comparing the square root of AVE values with inter-construct correlations, with all square root values exceeding corresponding correlations, indicating that each construct captures unique variance distinct from other constructs in the model.

### 4.3 Structural model and hypothesis testing

This section tests the research hypotheses and explores the impact of HRM practices on WFB and its indirect effects through mediating variables using structural equation modeling (SEM). Model fit indices and path analysis results are presented in Tables 3–5.

**Table 3:** Fitting indicators of SEM.

Fitting indicator	Value	Recommended value	Result
χ^2/df	2.45	< 3	Good
Root Mean Square Error of Approximation (RMSEA)	0.05	< 0.08	Good
Comparative Fit Index (CFI)	0.96	> 0.95	Good
Tucker-Lewis Index (TLI)	0.95	> 0.95	Good
Standardized Root Mean Square Residual (SRMR)	0.04	< 0.08	Good

**Table 4:** Analysis results of direct paths.

Path	Standardization coefficient (*β*)	Significance	Conclusion
Leadership → WFB	0.32	*p* < 0.001	Significant
PM → WFB	−0.15	*p* < 0.05	Significant
POS → WFB	0.45	*p* < 0.001	Significant
work pressure → WFB	−0.37	*p* < 0.001	Significant

**Table 5:** Analysis results of indirect paths (through mediating variables).

Indirect path	Standardized mediating effect	Significance	Conclusion
Leadership → ES → WFB	0.18	*p* < 0.001	Significant
PM → POS → WFB	0.12	*p* < 0.05	Significant
work pressure → ES → WFB	−0.22	*p* < 0.001	Significant

Results in Table 3 indicate that fitting indicators of SEM are within acceptable ranges. A chi-square to degrees of freedom ratio (χ^2^/df = 2.45) indicates parsimonious fit, while an RMSEA value of 0.05 suggests close model fit according to established guidelines (Hu and Bentler, 1999). CFI (0.96) and TLI (0.95) values exceeding 0.95 provide evidence of excellent model fit, and an SRMR value of 0.04 indicates minimal discrepancy between observed and predicted correlations. These indices collectively suggest that the theoretical model adequately represents the observed data.

Table 4 presents the direct effects of HRM practices on WFB. Leadership demonstrates a significant positive effect on WFB (*β* = 0.32, *p* < 0.001), supporting Hypothesis 1. This finding aligns with Li et al.'s (2017) comprehensive review of the leadership-work-family interface and provides empirical support for the theoretical proposition that supportive leadership facilitates employees’ boundary management capacity. Leaders who demonstrate concern for employees’ personal circumstances, provide schedule flexibility, and model healthy work-life integration enable their subordinates to more effectively navigate the competing demands of work and family roles. POS also exhibits a strong positive effect on WFB (*β* = 0.45, *p* < 0.001), representing the largest positive effect in the model. This finding indicates that employees who perceive greater organizational support are significantly better able to balance work and family demands. When organizations demonstrate genuine concern for employees’ well-being through family-friendly policies, flexible arrangements, and tangible support resources, employees develop confidence in their ability to meet both work and family obligations without compromising either domain. In contrast, PM shows a modest negative direct effect on WFB (*β* = −0.15, *p* < 0.05), suggesting that intensive performance evaluation may create additional stress that impairs work-family balance. Performance systems that emphasize continuous monitoring, frequent evaluation, and high-stakes accountability may generate anxiety and cognitive preoccupation that depletes psychological resources needed for effective boundary management. Work pressure demonstrates the strongest negative effect (*β* = −0.37, *p* < 0.001), confirming that job demands significantly undermine employees’ capacity to maintain equilibrium across life domains. Excessive workload, time pressure, and role overload consume psychological and physical resources that are essential for managing family responsibilities, consistent with COR theory predictions regarding resource depletion processes.

Table 5 reveals the mediation effects in the model. An indirect effect of leadership on WFB through ES is significant (*β* = 0.18, *p* < 0.001), with the total effect of leadership comprising both direct (*β* = 0.32) and indirect (*β* = 0.18) components, yielding a total effect of 0.50. This mediation effect accounts for approximately 36% of the total effect, indicating that employee satisfaction serves as a crucial psychological mechanism connecting leadership to work-family outcomes. According to COR theory, positive work experiences under supportive leadership generate psychological resources that can be invested in the family domain (Halbesleben et al., 2014). When employees feel satisfied with their jobs, they develop positive emotional states and cognitive resources that buffer against the depleting effects of work–family conflict. PM → POS → WFB pathway is also significant (*β* = 0.12, *p* < 0.05), suggesting that while PM has a negative direct effect, it can indirectly promote WFB by enhancing employees’ perceptions of organizational support when implemented fairly and developmentally. This suppression effect highlights the importance of considering both direct and mediated pathways when evaluating HRM practice effectiveness. Work pressure demonstrates a significant negative indirect effect through ES (*β* = −0.22, *p* < 0.001), highlighting that excessive work demands deplete psychological resources, reduce job satisfaction, and consequently impair work-family balance. Combined direct and indirect effects of work pressure (total effect = −0.59) underscore its substantial detrimental impact on employees’ capacity to balance competing life demands.

To examine whether these relationships vary across organizational contexts, subgroup analyses were conducted using data from four industries: manufacturing, services, information technology, and finance. Results are presented in Table 6.

**Table 6:** Subgroup analysis results by industry sector.

Industry	Leadership →WFB (*β*)	PM → WFB (*β*)	POS → WFB (*β*)	Work pressure →WFB (*β*)
Manufacturing	0.35 (*p* < 0.001)	−0.20 (*p* < 0.05)	0.48 (*p* < 0.001)	−0.40 (*p* < 0.001)
Services	0.30 (*p* < 0.001)	−0.18 (*p* < 0.05)	0.42 (*p* < 0.001)	−0.35 (*p* < 0.001)
Information technology	0.38 (*p* < 0.001)	−0.15 (*p* < 0.05)	0.50 (*p* < 0.001)	−0.38 (*p* < 0.001)
Finance	0.28 (*p* < 0.001)	−0.17 (*p* < 0.05)	0.40 (*p* < 0.001)	−0.33 (*p* < 0.001)

Table 6 reveals significant differences in HRM practices’ impact on WFB across sectors. Leadership has the strongest impact in IT (*β* = 0.38) but is smaller in finance (*β* = 0.28), reflecting IT work’s unique characteristics including project-based structures, longer hours, and blurred work-home boundaries. POS demonstrates consistently strong effects across all industries, strongest in IT (*β* = 0.50). PM shows more pronounced negative effects in manufacturing (*β* = −0.20), reflecting more rigid evaluation systems. Work pressure effects are strongest in manufacturing (*β* = −0.40), consistent with physically demanding work and shift schedules. These variations underscore the importance of tailoring HRM interventions to specific organizational contexts.

This study uses AMOS software to fit an extended SEM model incorporating comprehensive HRM practices and their pathways to WFB. Standardized path coefficients for the extended model are presented in Table 7. Model fit indices are good (χ^2^/df = 2.35, RMSEA = 0.05, CFI = 0.96, TLI = 0.95, SRMR = 0.04), suggesting that the model effectively explains the relationships in the data. Results indicate that HRM practices directly or indirectly promote WFB by enhancing ES and POS while reducing work pressure. Specifically, HRM practices have strong effects on ES (*β* = 0.56) and POS (*β* = 0.45), indicating that comprehensive HRM approaches effectively enhance both employee psychological well-being and perceptions of organizational support.

**Table 7:** The standardized path coefficient of the expanded SEM model.

Path	Standardized path coefficient (*β*)	Significance (*p*)
HRMP → ES	0.56 (*p* < 0.001)	Significant
HRMP → POS	0.45 (*p* < 0.001)	Significant
HRMP → Work pressure	−0.37 (*p* < 0.001)	Significant
HRMP → POSus	0.40 (*p* < 0.001)	Significant
HRMP → EFB	0.35 (*p* < 0.001)	Significant
ES → WFB	0.45 (*p* < 0.001)	Significant
POS → WFB	0.39 (*p* < 0.001)	Significant
Work pressure → WFB	−0.37 (*p* < 0.001)	Significant
POSus → EFB	0.50 (*p* < 0.001)	Significant
EFB → WFB	0.25 (*p* < 0.01)	Significant

Extended model results confirm that comprehensive HRM practices operate through multiple pathways to influence WFB. Leadership demonstrates a particularly strong effect on ES (*β* = 0.48), suggesting that supportive leadership behaviors are especially effective at enhancing employee psychological well-being. PM shows a significant positive relationship with POS (*β* = 0.32) when implemented developmentally, indicating that fair and growth-oriented performance systems can strengthen employees’ sense of organizational support. ES and POS both have substantial direct effects on WFB (*β* = 0.45 and *β* = 0.39, respectively), while work pressure maintains its significant negative impact (*β* = −0.37). These findings validate the dual-pathway model and highlight the interconnected nature of HRM practices in shaping work-family outcomes, demonstrating that organizations can enhance WFB through both resource-building and stress-reduction strategies.

**Figure 8:**
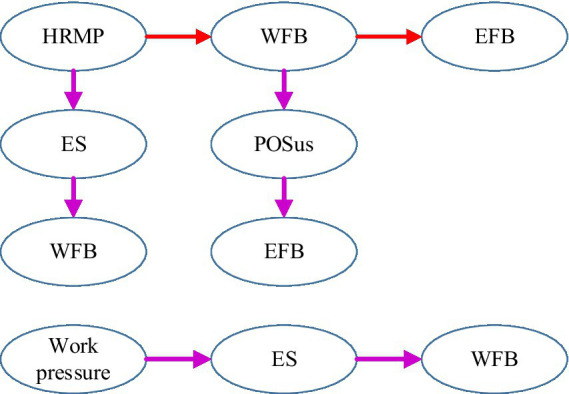
The comprehensive impact path of HRMP on WFB and sustainability outcomes.

Figure 8 illustrates comprehensive impact paths of HRM practices on WFB. This model demonstrates that leadership and POS function as resource-building mechanisms enhancing WFB, while work pressure operates as a resource-depleting factor. ES and POS serve as critical mediating mechanisms transmitting HRM practice effects to work-family outcomes, consistent with COR theory and boundary theory. Direct effects represent immediate impacts on boundary management capacity, while indirect effects capture psychological resource accumulation processes through which practices influence outcomes over time.

## 5 Discussion

### 5.1 Theoretical implications

This study makes several contributions to the work–family literature by integrating boundary theory and COR theory to examine dual psychological pathways linking HRM practices to work-family balance. Through SEM analysis, intrinsic links between HR practices and work-family balance indicators are revealed, which resonates with the study by Danilwan and Dirhamsyah (2022) on HRM and organizational performance. Findings demonstrate that leadership behavior exerts both direct and indirect effects on WFB. A direct effect (*β* = 0.32) is notably larger than the average correlation of r = 0.21 reported in Li et al.'s (2017) meta-analysis. This larger effect size may reflect our East Asian sample’s cultural context, where hierarchical relationships and paternalistic leadership traditions amplify supervisor support importance. In Confucian cultures, employees often expect leaders to extend concern beyond work performance to include family well-being (Phelan, 2023), intensifying the impact of supportive leadership on work-family outcomes.

Identification of ES as a significant mediator demonstrates that employee satisfaction serves as a crucial psychological mechanism connecting leadership to work-family outcomes. According to COR theory, positive work experiences under supportive leadership generate psychological resources—including positive affect, self-efficacy, and energy—that can be invested in the family domain (Halbesleben et al., 2014). Qualitative interviews corroborated this mechanism, with employees frequently noting that supervisors’ understanding of family responsibilities made a substantial difference in their ability to manage conflicts. Partial mediation suggests leadership influences WFB through multiple pathways, including direct flexibility provision, modeling of healthy work-life integration, and creation of supportive work norms that legitimize family responsibilities.

Finding that PM has a negative direct effect on WFB while simultaneously enhancing POS reveals the complex, dual-edged nature of performance management in organizational settings. This suppression effect—where PM shows opposing direct and indirect effects—has important theoretical implications. When PM systems emphasize developmental feedback, clear expectations, and fair processes, they signal organizational investment in employees, thereby enhancing POS. However, intensive monitoring and evaluation may directly create stress undermining WFB. This pattern suggests that design and implementation of PM systems critically determine whether net effects on WFB are positive or negative. Organizations should design performance management approaches demonstrating care for employee development while minimizing evaluative stress.

### 5.2 Practical implications

Findings offer several actionable recommendations for organizational practice. First, organizations should invest in leadership development programs enhancing supervisors’ awareness and skills in supporting WFB. Training should emphasize demonstrating genuine concern for employees’ circumstances, providing schedule flexibility, and modeling healthy work-life integration. Strong leadership effects in IT (*β* = 0.38) underscore the strategic importance of supervisory support in high-demand environments. Leaders should be trained to recognize work–family conflict signs and proactively offer support through schedule adjustments and workload management.

Second, organizations should redesign PM systems to emphasize developmental rather than purely evaluative functions. PM practices incorporating constructive feedback, recognizing work-family challenges, and allowing flexibility can enhance POS while minimizing negative WFB effects. Industry variations suggest that manufacturing and technology sectors may particularly benefit from more flexible approaches. Performance evaluations could incorporate WFB indicators assessing sustainable work patterns and team support behaviors.

Third, enhancing POS through family-friendly policies and flexible work arrangements represents a high-leverage intervention given strong POS effects on WFB across all sectors (*β* = 0.40–0.50). Organizations should implement comprehensive initiatives including flexible scheduling, remote work options, parental leave policies exceeding statutory minimums, and dependent care assistance programs. Consistent strength of POS effects across industries suggests organizational support investments yield WFB benefits regardless of sector characteristics.

This study has compiled a detailed practice summary (Table 8) to help organizations better understand and implement work-family supportive HRM practices. It covers specific measures in leadership development, performance management, work arrangements, and employee engagement, along with expected outcomes and implementation recommendations for each practice area.

**Table 8:** Summary of SHRMP.

Practice areas	Specific measure	Expected effect	Implementation suggestion
Leadership development	1. Provide specialized training on sustainable development; 2. Set clear sustainable development goals; 3. Leaders lead by example; 4. Establish incentive mechanisms.	Enhance leaders’ awareness and sense of responsibility toward sustainable development; Promote the transformation of organizational culture toward sustainable development; Motivate leaders to actively practice environmental protection concepts.	Regularly hold training courses and seminars; Incorporate sustainable development goals into annual plans; Leaders take the lead in reducing resource waste.
Optimization of the PM system	1. Establish “green performance” indicators; 2. Regular evaluation and feedback; 3. Provide rewards and recognition; 4. Encourage cross departmental collaboration	Promote employees to pay attention to environmental protection and social responsibility in their work; Enhance employees’ environmental awareness and actions; Strengthen collaboration and innovation among departments.	Add environmental indicators to performance evaluation; Conduct quarterly evaluations and provide feedback; Establish the title of “Environmental Star”; Organize cross departmental project teams.
Flexible working arrangements	1. Promote remote work; 2. Provide flexible working hours; 3. Shared office space; 4. Offer green commuting solutions.	Reduce commuting time and carbon emissions; Improve employees’ job satisfaction and quality of life; Reduce energy consumption in office space.	Provide necessary technical support; Allow employees to choose their commuting time; Collaborate with other companies to share office space; Provide transportation subsidies.
Employee engagement and communication	1. Regularly organize “Green Forums”; 2. Establish an “Environmental Proposal Competition”; 3. Create a “Green Ambassador” team; 4. Implement a transparent communication mechanism.	Enhance employees’ environmental awareness and sense of responsibility; Inspire employees’ creativity and sense of participation; Improve employees’ sense of belonging and satisfaction.	Hold forums monthly or quarterly; Establish a review committee to select outstanding proposals; Select employees with high enthusiasm for environmental protection as green ambassadors.

### 5.3 Cultural context and boundary conditions

This study was conducted in East Asian contexts (China, Korea, and Thailand), where Confucian cultural values significantly shape workplace dynamics and work-family relationships. It is crucial to consider how these cultural characteristics may have influenced our findings. In collectivistic cultures, leadership behaviors emphasizing group harmony, interpersonal relationships, and employee welfare may be particularly effective in promoting work-family balance. Park et al. (2023) found that supportive leadership had stronger effects in Korea compared to Western contexts, aligning with our larger-than-typical leadership effects. Cultural value placed on hierarchical relationships and paternalistic care likely intensifies leadership support impact.

A modest negative direct effect of PM on WFB (*β* = −0.15) may reflect cultural dynamics specific to East Asian contexts, where performance systems emphasizing individual accountability may conflict with group harmony and collective achievement values, potentially creating stress spilling over into family life. However, when PM is implemented demonstrating organizational investment in employee development, negative effects can be partially offset. Shimazu et al.'s (2023) randomized controlled trial among Japanese dual-earner couples found that culturally-tailored interventions addressing role conflicts were more effective than generic programs, highlighting the importance of cultural adaptation in HRM practices.

While our theoretical framework (Boundary Theory and Conservation of Resources Theory) provides universal principles for understanding work-family dynamics, effect magnitudes and specific mechanisms may vary across cultural contexts. We propose hypotheses for cross-cultural testing: First, in more individualistic Western cultures, formal work-family policies and structural support may play larger roles relative to interpersonal leadership relationships. Second, in cultures with stronger work-family boundaries, the mediating role of ES may be less pronounced because psychological spillover between domains is less prevalent. Third, in contexts with different gender role expectations, HRM practice effects may differ systematically for men and women.

We acknowledge that our sample is limited to East Asian contexts. Cultural homogeneity limits generalizability to different value systems. Future research should test our model in Western and other non-Western contexts to establish boundary conditions. Multi-country comparative studies using cultural dimensions as moderators would be valuable for understanding contextual factors shaping HRM-WFB relationships.

In conclusion, this study validates direct effects of HRM practices on WFB and reveals critical mediating roles of ES and POS. Findings underscore the importance of cultivating supportive leadership, designing humane PM systems, and enhancing POS when formulating HRM strategies. Integration of boundary theory and COR theory provides a robust framework for understanding these relationships, while identification of mediating mechanisms offers actionable targets for organizational interventions.

## 6 Conclusion

This study advances understanding of the psychological mechanisms linking human resource management practices to work-family balance by integrating boundary theory and conservation of resources theory within a unified empirical framework. Through structural equation modeling with data from 469 employees across diverse industries, we demonstrate that leadership behavior exerts both direct (*β* = 0.32, *p* < 0.001) and indirect effects on work-family balance through employee satisfaction, which mediates approximately 36% of the total effect. Furthermore, the findings reveal that performance management practices influence work-family balance through a distinct pathway via perceived organizational support (*β* = 0.39, *p* < 0.001), while simultaneously exhibiting a negative direct effect that reflects the dual-edged nature of intensive evaluation systems. These differential mediation pathways contribute to the work–family literature by elucidating how distinct HRM practices operate through complementary psychological mechanisms—affective responses versus cognitive attributions—to shape employees’ capacity for boundary management.

The practical implications of these findings are substantial for organizational decision-makers seeking to enhance employee work-family balance. Specifically, organizations should prioritize leadership development programs that cultivate supervisors’ awareness and skills in supporting work-family integration, given the robust direct and indirect effects of supportive leadership observed across industry sectors. Additionally, the counterbalancing effects of performance management suggest that organizations should redesign evaluation systems to emphasize developmental feedback and recognition of work-family challenges, thereby strengthening perceived organizational support while minimizing the stress-inducing consequences of intensive monitoring.

Despite these contributions, several limitations warrant acknowledgment. The cross-sectional research design precludes definitive causal inferences regarding the temporal relationships among variables, although our theoretical framework provides strong conceptual grounding for the proposed pathways. Moreover, reliance on self-reported questionnaire data, despite procedural remedies for common method bias, may introduce measurement artifacts that influence parameter estimates. The study’s East Asian sample context also limits generalizability to Western organizational settings where cultural values regarding hierarchy and work-family boundaries differ substantially. Future research should employ multi-wave longitudinal designs to establish temporal precedence, incorporate multi-source data collection including supervisor ratings and objective organizational records, and conduct cross-cultural comparative studies to identify boundary conditions moderating the HRM-work-family balance relationship across diverse cultural contexts.

## Data Availability

The original contributions presented in the study are included in the article/supplementary material, further inquiries can be directed to the corresponding authors.

## References

[ref1] Allen T. D. Cho E. Meier L. L. (2014). Work–family boundary dynamics. Annu. Rev. Organ. Psychol. Organ. Behav. 1, 99–121. doi: 10.1146/annurev-orgpsych-031413-091330

[ref2] Allen T. D. Herst D. E. Bruck C. S. Sutton M. (2000). Consequences associated with work-to-family conflict: a review and agenda for future research. J. Occup. Health Psychol. 5, 278–308. doi: 10.1037/1076-8998.5.2.278, 10784291

[ref3] Anderson J. C. Gerbing D. W. (1988). Structural equation modeling in practice: a review and recommended two-step approach. Psychol. Bull. 103, 411–423. doi: 10.1037/0033-2909.103.3.411

[ref4] Ashforth B. E. Kreiner G. E. Fugate M. (2000). All in a day's work: boundaries and micro role transitions. Acad. Manag. Rev. 25, 472–491. doi: 10.5465/amr.2000.3363315

[ref5] Bagger J. Li A. (2014). How does supervisory family support influence employees' attitudes and behaviors? A social exchange perspective. J. Manage. 40, 1123–1150. doi: 10.1177/0149206311413922

[ref6] Bankins S. Ocampo A. C. Marrone M. Restubog S. L. D. Woo S. E. (2024). A multilevel review of artificial intelligence in organizations: implications for organizational behavior research and practice. J. Organ. Behav. 45, 159–182. doi: 10.1002/job.2735

[ref7] Baral R. Bhargava S. (2010). Work-family enrichment as a mediator between organizational interventions for work-life balance and job outcomes. J. Manag. Psychol. 25, 274–300. doi: 10.1108/02683941011023749

[ref8] Baruch Y. Holtom B. C. (2008). Survey response rate levels and trends in organizational research. Hum. Relat. 61, 1139–1160. doi: 10.1177/0018726708094863

[ref001] Bass B. M. (1985). Leadership: Good, better, best. Organizational Dynamics. 13, 26–40. doi: 10.1016/0090-2616(85)90028-2

[ref9] Bass B. M. Avolio B. J. (1997). Full range leadership development: Manual for the multifactor leadership questionnaire. Redwood City, CA: Mind Garden.

[ref10] Bellmann L. Hübler O. (2021). Working from home, job satisfaction and work-life balance—robust or heterogeneous links? Int. J. Manpow. 42, 424–441. doi: 10.1108/IJM-10-2019-0458

[ref11] Braun V. Clarke V. (2006). Using thematic analysis in psychology. Qual. Res. Psychol. 3, 77–101. doi: 10.1191/1478088706qp063oa

[ref12] Brummelhuis L. L. Bakker A. B. (2012). A resource perspective on the work-home interface: the work-home resources model. Am. Psychol. 67, 545–556. doi: 10.1037/a0027974, 22506688

[ref13] Clark S. C. (2000). Work/family border theory: a new theory of work/family balance. Hum. Relat. 53, 747–770. doi: 10.1177/0018726700536001

[ref14] Creswell J. W. Creswell J. D. (2018). Research design: Qualitative, quantitative, and mixed methods approaches. London: Sage Publications.

[ref15] Danilwan Y. Dirhamsyah D. (2022). Linking human resource management practices to organizational performance: evidence from Indonesian higher education. Cogent Bus. Manag. 9:2114304. doi: 10.1080/23311975.2022.2114304

[ref16] Daraba D. Wirawan H. Salam R. Faisal M. (2021). Working from home during the corona pandemic: investigating the role of authentic leadership, psychological capital, and gender on employee performance. Cogent Bus. Manag. 8:1885573. doi: 10.1080/23311975.2021.1885573

[ref17] Deci E. L. Ryan R. M. (2000). The "what" and "why" of goal pursuits: human needs and the self-determination of behavior. Psychol. Inq. 11, 227–268. doi: 10.1207/S15327965PLI1104_01

[ref18] DeNisi A. S. Murphy K. R. (2017). Performance appraisal and performance management: 100 years of progress? J. Appl. Psychol. 102, 421–433. doi: 10.1037/apl0000085, 28125265

[ref19] DeNisi A. S. Pritchard R. D. (2006). Performance appraisal, performance management and improving individual performance: a motivational framework. Manag. Organ. Rev. 2, 253–277. doi: 10.1111/j.1740-8784.2006.00042.x

[ref20] Doty D. H. Glick W. H. (1998). Common methods bias: does common methods variance really bias results? Organ. Res. Methods 1, 374–406. doi: 10.1177/109442819814002

[ref21] Eisenberger R. Huntington R. Hutchison S. Sowa D. (1986). Perceived organizational support. J. Appl. Psychol. 71, 500–507. doi: 10.1037/0021-9010.71.3.500

[ref22] Frone M. R. Russell M. Cooper M. L. (1992). Antecedents and outcomes of work-family conflict: testing a model of the work-family interface. J. Appl. Psychol. 77, 65–78. doi: 10.1037/0021-9010.77.1.65, 1556042

[ref23] Golden T. D. Fromen A. (2011). Does it matter where your manager works? Comparing managerial work mode (traditional, telework, virtual) across subordinate work experiences and outcomes. Hum. Relat. 64, 1451–1475. doi: 10.1177/0018726711418387

[ref24] Grewal R. Cote J. A. Baumgartner H. (2004). Multicollinearity and measurement error in structural equation models: implications for theory testing. Mark. Sci. 23, 519–529. doi: 10.1287/mksc.1040.0070, 19642375

[ref25] Guest G. Bunce A. Johnson L. (2006). How many interviews are enough? An experiment with data saturation and variability. Field Methods 18, 59–82. doi: 10.1177/1525822X05279903

[ref26] Halbesleben J. R. B. Neveu J.-P. Paustian-Underdahl S. C. Westman M. (2014). Getting to the "COR": understanding the role of resources in conservation of resources theory. J. Manage. 40, 1334–1364. doi: 10.1177/0149206314527130

[ref27] Hauff S. Alewell D. Kattenbach R. (2022). Telework and work-family conflict across workplaces: investigating the implications of work-family-supportive and performance-oriented HR practices. Int. J. Hum. Resour. Manag. 33, 4498–4525. doi: 10.1080/09585192.2022.2063066

[ref28] Hobfoll S. E. (1989). Conservation of resources: a new attempt at conceptualizing stress. Am. Psychol. 44, 513–524. doi: 10.1037/0003-066X.44.3.513, 2648906

[ref29] Hobfoll S. E. (2001). The influence of culture, community, and the nested-self in the stress process: advancing conservation of resources theory. Appl. Psychol. 50, 337–421. doi: 10.1111/1464-0597.00062

[ref30] Hu L. T. Bentler P. M. (1999). Cutoff criteria for fit indexes in covariance structure analysis: conventional criteria versus new alternatives. Struct. Equ. Model. Multidiscip. J. 6, 1–55. doi: 10.1080/10705519909540118

[ref31] Huang J. Wang Y. You X. (2021). The job demands-resources model and job burnout: the mediating role of personal resources. Curr. Psychol. 40, 295–305. doi: 10.1007/s12144-018-9926-7

[ref32] Isa M. F. M. Indrayati N. (2023). The influence of work-life balance and job satisfaction on employee performance. Int. J. Prof. Bus. Rev. 8:e01236. doi: 10.26668/businessreview/2023.v8i4.1236

[ref33] Kline R. B. (2016). Principles and practice of structural equation modeling. New York: Guilford Press.

[ref34] Kurtessis J. N. Eisenberger R. Ford M. T. Buffardi L. C. Stewart K. A. Adis C. S. (2017). Perceived organizational support: a meta-analytic evaluation of organizational support theory. J. Manage. 43, 1854–1884. doi: 10.1177/0149206315575554

[ref36] Li A. Shaffer J. Bagger J. (2017). The psychological well-being of disability caregivers: examining the roles of family strain, family-to-work conflict, and perceived supervisor support. J. Occup. Health Psychol. 22, 98–114. doi: 10.1037/ocp0000027, 25181282

[ref37] Locke E. A. (1976). “The nature and causes of job satisfaction” in Handbook of industrial and organizational psychology. ed. Dunnette M. D. (Chicago, IL: Rand McNally), 1297–1349.

[ref38] Locke E. A. Latham G. P. (2002). Building a practically useful theory of goal setting and task motivation: a 35-year odyssey. Am. Psychol. 57, 705–717. doi: 10.1037/0003-066X.57.9.705, 12237980

[ref39] Maertz C. P. Boyar S. L. (2011). Work-family conflict, enrichment, and balance under "levels" and "episodes" approaches. J. Manage. 37, 68–98. doi: 10.1177/0149206310382455

[ref40] Na-Nan K. Chaiprasit K. Pukkeeree P. (2020). Factor analysis-validated comprehensive employee job performance scale. Int. J. Qual. Reliab. Manag. 37, 30–45. doi: 10.1108/IJQRM-06-2018-0173

[ref41] Netemeyer R. G. Boles J. S. McMurrian R. (1996). Development and validation of work–family conflict and family–work conflict scales. J. Appl. Psychol. 81, 400–410. doi: 10.1037/0021-9010.81.4.400

[ref42] Nippert-Eng C. E. (1996). Home and work: Negotiating boundaries through everyday life. Chicago, IL: University of Chicago Press.

[ref43] Park Y. Kim J. Lee H. (2023). The influences of supportive leadership and family social support on female managers' organizational effectiveness: the mediating effect of positive spillover between work and family. Behav. Sci. 13:639. doi: 10.3390/bs13080639, 37622779 PMC10451751

[ref44] Patton M. Q. (2015). Qualitative research and evaluation methods. London: Sage Publications.

[ref45] Petitta L. Probst T. M. Ghezzi V. Barbaranelli C. (2024). Job insecurity and work-family interface as predictors of mental and physical health: the moderating role of family-work stereotype threat. J. Occup. Organ. Psychol. 97, 452–478. doi: 10.1111/joop.12478, 41455825

[ref46] Phelan J. (2023). “Harnessing cultural intelligence in cross-cultural business and management” in Handbook of research on cross-culture business and management. ed. Kenney C. (Pennsylvania: IGI Global), 59–88.

[ref47] Podsakoff P. M. MacKenzie S. B. Lee J.-Y. Podsakoff N. P. (2003). Common method biases in behavioral research: a critical review of the literature and recommended remedies. J. Appl. Psychol. 88, 879–903. doi: 10.1037/0021-9010.88.5.879, 14516251

[ref48] Podsakoff P. M. MacKenzie S. B. Podsakoff N. P. (2012). Sources of method bias in social science research and recommendations on how to control it. Annu. Rev. Psychol. 63, 539–569. doi: 10.1146/annurev-psych-120710-100452, 21838546

[ref49] Podsakoff P. M. Organ D. W. (1986). Self-reports in organizational research: problems and prospects. J. Manage. 12, 531–544. doi: 10.1177/014920638601200408

[ref50] Preacher K. J. Hayes A. F. (2008). Asymptotic and resampling strategies for assessing and comparing indirect effects in multiple mediator models. Behav. Res. Methods 40, 879–891. doi: 10.3758/BRM.40.3.879, 18697684

[ref52] Schafer J. L. Graham J. W. (2002). Missing data: our view of the state of the art. Psychol. Methods 7, 147–177. doi: 10.1037/1082-989X.7.2.147, 12090408

[ref53] Shimazu A. Fujiwara T. Iwata N. Kato Y. Kawakami N. Maegawa N. . (2023). Effects of work-family life support program on the work-family interface and mental health among Japanese dual-earner couples with a preschool child: a randomized controlled trial. J. Occup. Health 65:e12397. doi: 10.1002/1348-9585.12397, 37017650 PMC10075245

[ref54] Smith P. C. Kendall L. M. Hulin C. L. (1969). The measurement of satisfaction in work and retirement: A strategy for the study of attitudes. Chicago, IL: Rand McNally.

[ref55] Stollberger J. Las Heras M. Rofcanin Y. Bosch M. J. (2019). Serving followers and family? A trickle-down model of how servant leadership shapes employee work performance. J. Vocat. Behav. 112, 158–171. doi: 10.1016/j.jvb.2019.02.003

[ref56] Vidè F. Micacchi L. Barbieri M. Valotti G. (2023). The renaissance of performance appraisal: engaging public employees through perceived developmental purpose and justice. Rev. Public Pers. Adm. 43, 623–651. doi: 10.1177/0734371X221116584

[ref57] Wan M. Shaffer M. A. Singh R. Zhang Y. (2022). Spoiling for a fight: a relational model of daily work-family balance satisfaction. J. Occup. Organ. Psychol. 95, 60–89. doi: 10.1111/joop.12368

[ref58] Wayne J. H. Matthews R. Crawford W. Casper W. J. (2020). Predictors and processes of satisfaction with work-family balance: examining the role of personal, work, and family resources and conflict and enrichment. Hum. Resour. Manag. 59, 25–42. doi: 10.1002/hrm.21971

[ref59] Williams L. J. Cote J. A. Buckley M. R. (1989). Lack of method variance in self-reported affect and perceptions at work: reality or artifact? J. Appl. Psychol. 74, 462–468. doi: 10.1037/0021-9010.74.3.462

[ref60] Williams E. A. McCombs K. M. (2023). Understanding employee work-life conflict experiences: self-leadership responses involving resource management for balancing work, family, and professional development. J. Occup. Organ. Psychol. 96, 807–827. doi: 10.1111/joop.12451

